# Feasibility of gabapentin as an intervention for neurorecovery after an acute spinal cord injury: Protocol

**DOI:** 10.3389/fneur.2022.1033386

**Published:** 2022-11-07

**Authors:** James R. Wilson, Samuel Doty, Jordan C. Petitt, Mohamed El-Abtah, John J. Francis, Megan G. Sharpe, Michael L. Kelly, Kim D. Anderson

**Affiliations:** ^1^MetroHealth Rehabilitation Institute, MetroHealth System, Cleveland, OH, United States; ^2^Department of Physical Medicine and Rehabilitation, Case Western Reserve University School of Medicine, Cleveland, OH, United States; ^3^Department of Neurological Surgery, Case Western Reserve University School of Medicine, Cleveland, OH, United States; ^4^MetroHealth Medical Center, MetroHealth System, Cleveland, OH, United States

**Keywords:** neurorecovery, dose exploration, spinal cord injury, gabapentin, traumatic

## Abstract

**Introduction:**

This protocol is describing the first ever prospective, mock-efficacy, dose exploration trial design testing the feasibility of administering gabapentin in the acute setting as an intervention for neurorecovery. Gabapentin is an FDA-approved medication for treating seizures and postherpetic neuralgia and is used broadly off-label for neuropathic pain management for many conditions, including spinal cord injury. Emerging data suggests that when given early after spinal cord injury onset and in low-medium doses, gabapentin may have properties that promote recovery of neurological function. The objective of this trial is to assess the feasibility of conducting an efficacy trial in which gabapentin is started early after injury, is restricted in its dose, and is not used for pain management.

**Methods and analysis:**

Forty-two people aged 18 years or older with any level and any severity of spinal cord injury induced by a trauma will be enrolled, randomized, and have the first dose of study medication by 120 h post-injury onset. Participants will be randomly assigned to one of three groups: 600, 1,800 mg/day gabapentin, or placebo. Study medication will be given for a 90-day duration. Blinded assessments will be obtained at 7 days post-injury (baseline), 30 days post-injury (interim), after the 90-day treatment duration/approximately 3 months post-injury (end of treatment), and at 6 months post-injury (end of study). The key analysis parameters will evaluate feasibility of recruitment of target population, delivery of drug treatment protocol, maintenance of blinding, and retention of participants.

**Discussion:**

Outputs from this trial will inform research and clinical practice on the effects of manipulating gabapentin for non-pain management purposes in the acute setting and will guide the development of a properly powered efficacy trial of gabapentin as an intervention for neurorecovery in spinal cord injury.

**Ethics and dissemination:**

The study was approved by the MetroHealth Institutional Review Board (IRB21-00609) and registered at clinicaltrials.gov prior to enrolling any participants. Dissemination will include peer-reviewed publications, presentations at professional conferences and in the community, and through other healthcare and public venues.

**Clinical trial registration:**

www.ClinicalTrials.gov, identifier: NCT05302999; protocol version 1.1 approved 05/23/2022.

**Trial funding:**

National Institute on Disability, Independent Living and Rehabilitation Research.

## Introduction

Gabapentin is an FDA-approved medication initially indicated for the treatment of seizures. Its action as an anticonvulsant is related to its ability to bind to the α2δ subunits of voltage-sensitive calcium channels (1). In addition, gabapentin has been approved for post-herpetic neuralgia, based on a multi-center randomized clinical trial reporting decreased average daily pain scores after an 8-week treatment period with the drug (2). It is thought that this analgesic mechanism is a result of astrocyte-derived thrombospondins promoting excitatory synapse formation via α2δ1 subunits (3). Although gabapentin has only two FDA-approved indications, it has been prescribed off-label for many years for multiple reasons, including management of neuropathic pain in spinal cord injury (SCI) (4, 5).

Gabapentin is recommended as a first-line treatment for SCI-induced neuropathic pain (6). Administration of 1,800 mg/day has shown good levels of pain relief in neuropathic pain and is a common target dose to minimize side effects (7). Maximal doses tested in clinical trials for SCI-induced neuropathic pain have ranged up to 3,600 mg/day and the major adverse events reported were dizziness and somnolence (8–10). Other side effects that are recommended to monitor include peripheral edema, weakness, fatigue, nausea, diarrhea, constipation, blurred vision, headache, and dry mouth (11). There is emerging evidence, both in animals and in humans, that gabapentin may also have a role in neurorecovery when administered early after SCI.

In rodent models of SCI, it has been shown that a single dose of gabapentin delivered 2–3 weeks post-injury resulted in a significant decrease in both spastic behavior and electromyography activity when compared to control animals (12) as well as a reduction in autonomic dysreflexia (AD) and tail spasticity in response to colorectal distension (13). Daily administration of low-dose (human equivalent dose of 648 mg/day) gabapentin for the first four weeks post-SCI in rats further suggests a role in mitigating evoked AD (14); high-dose (human equivalent dose of 5,184 mg/day) gabapentin did not have this effect (14). Additionally, it was recently demonstrated in a mouse model that daily administration of a low-medium dose (human equivalent dose of 1,296 mg/day) of gabapentin for 5 weeks post-SCI resulted in the blockade of excitatory synaptogenesis and sprouting of autonomic fibers innervating immune organs as well as nociceptive fibers that trigger AD (4). Functionally, this was correlated with a reduction in the frequency of spontaneous AD and severity of evoked AD as well as protection from SCI-induced immune suppression, which persisted for a month after stopping gabapentin (4). Daily administration of low-dose (human equivalent dose of 648 mg/day) gabapentin, started early after C5 hemisection in mice and continued for 4 months, has also been demonstrated to lead to sprouting of corticospinal tract fibers and improvement in forelimb function (15).

In humans, there is a growing body of evidence that gabapentin may have a positive effect on the amount of motor recovery as measured by the Total Motor Score (TMS) of the International Standards for Neurological Classification of SCI (ISNCSCI). This is based on retrospective analysis of the prospective European Multicenter SCI (EMSCI) observational study database. It was initially discovered, while performing an analysis related to pain, that individuals in the database who had been administered anticonvulsant medication within the 1st month post-injury gained an average of 7.3 additional TMS points at 1 year when compared to individuals who had not been administered anticonvulsant medication within the 1st month post-SCI (16). That cohort included individuals with tetraplegia and paraplegia as well as all levels of severity. A larger analysis confirmed the gain in motor recovery when anticonvulsants were started in the 1st month after injury (average of 6.3 additional TMS points at 1 year compared to those that were not administered anticonvulsants) and identified that the most frequently administered anticonvulsants were gabapentinoids (17). A gain of an average of 9 motor points is roughly equivalent to an improvement of one spinal segment, which can translate to a significant functional impact (as measured by the Spinal Cord Independence Measure) when the gain is in the cervical or lumbosacral regions (18). Good functional assessments of the thoracic segments are still lacking. An analysis of the Sygen database, a large (*N* = 797), well-designed clinical trial conducted primarily before gabapentinoids came to the market, revealed that non-gabapentinoid anticonvulsants given within 1-month post-SCI had no effect on total motor scores (19). Most recently, analysis of the EMSCI database enabled the longitudinal modeling of drug-by-time effect sizes on TMS point recovery with the largest effect size being when gabapentiniods were administered within the first 5 days following SCI (20). Interpretation of this effect size must be taken with caution, however, as it is based on a small number of cases (*n* = 14) and with no information regarding dose. To date, there have been no prospective studies evaluating early administration of gabapentin in humans with SCI.

### Objective

Overall, emerging preclinical and clinical evidence suggests that early initiation of low to medium doses of gabapentin and continued delivery for a range of 2 weeks to 4 months has a persistent, positive effect on motor and autonomic neurologic recovery. However, there are several questions that are important to understand before testing the efficacy of early administration of gabapentin as an intervention for neurorecovery. These revolve around the appropriate target population recruitment, the ability to deliver the treatment per protocol, the ability to maintain blinded assessors, and participant retention. The objective of the proposed study is to conduct the first ever prospective, dose-exploration trial to test the feasibility of early administration of gabapentin as an intervention for neurorecovery.

## Methods

### Trial design and setting

**Figure 1:**
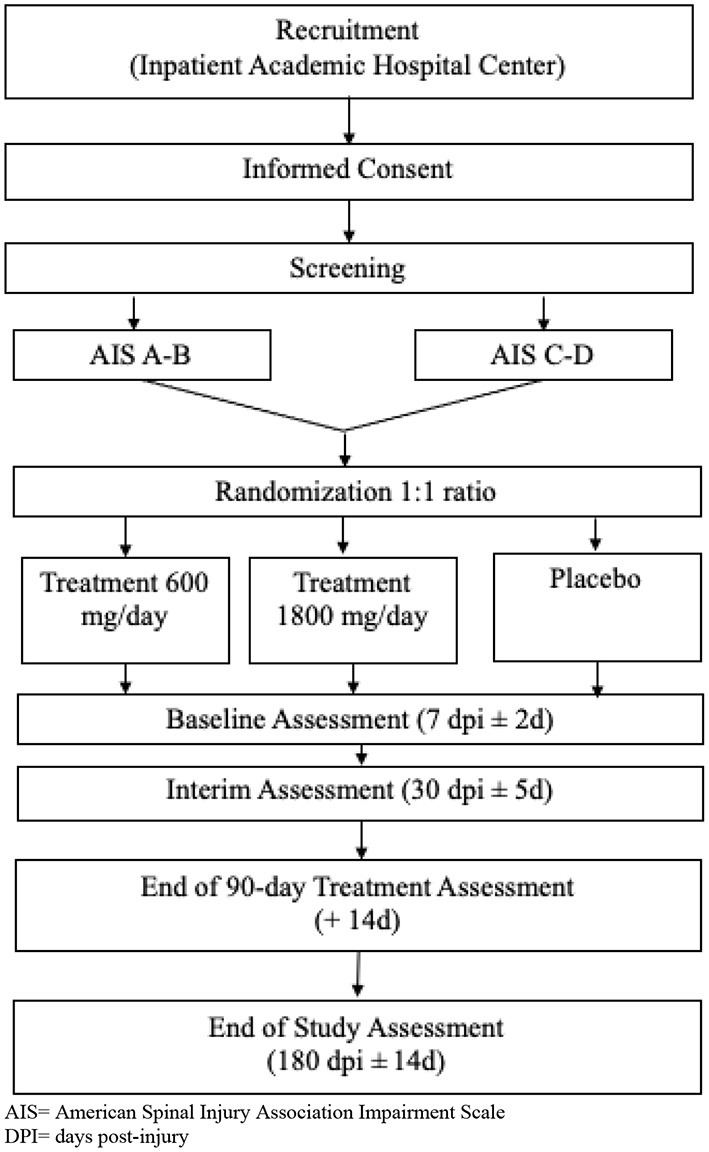
Study Flow Chart. AIS, American Spinal Injury Association Impairment Scale; DPI, days post-injury.

This is a prospective feasibility trial with randomized, controlled, and blinded features. To determine key issues regarding gabapentin dosing and restrictions, placebo use, and how those factors interplay with pain management, a mock efficacy design will be employed (Figure 1). The trial will feature a three-arm parallel group design, two arms receiving varying gabapentin doses, and the third arm receiving placebo. Placebo will be necessary for a future properly controlled efficacy trial. However, because gabapentin is used so ubiquitously for pain management, we need to understand the effect of withholding gabapentin for a 90-day period. Therefore, in the current study placebo is being administered primarily for feasibility reasons. This is a single-center study being conducted at an academic Level 1 trauma center in the USA that specializes in SCI acute care and rehabilitation.

### Participants and sample size

#### Eligibility criteria

Several of the inclusion criteria were intentionally kept broad because this is a study with key questions around enrollment and the protocol. Additionally, gabapentin has been used broadly and at much higher clinical doses in all levels and severities of SCI and there are no safety reasons to exclude specific injury levels or severities. Individuals will not be excluded based on race, ethnicity, or sex/gender. Individuals under the age of 18 will be excluded because this study will be the first prospective investigation of the safety and feasibility of administering gabapentin as a neurorestorative agent in adults with acute SCI.

Inclusion criteria:

1. Traumatic SCI
2. All levels of SCI
3. All severities of SCI, ASIA Impairment Scale (AIS) Grades A-D
4. Age 18 years and older
5. Agree to participate and start study drug within 120 h post-injury
6. Adequate cognition and communication to provide informed consent.

Exclusion criteria:

1. Presence of moderate/severe traumatic brain injury as defined by Glasgow Coma Score less than 13 at 120 h post-injury
2. Documented use of gabapentinoids at the time of injury.

#### Sample size

Studies analyzing feasibility are not intended to determine statistical significance, therefore traditional sample size calculations to power efficacy are inappropriate (21, 22). Rather, sample size should be large enough to yield useful data to answer the feasibility questions. There are several rules of thumb for determining a sample size for a feasibility study (typical range is 12–35 participants per arm; (23, 24). As described below, our most important neurologic outcome measure will be the ISNCSCI (all existing human data demonstrating effect of gabapentin on neurorecovery has been derived from the ISNCSCI). Differences in natural recovery due to injury severity could influence variability in ISNCSCI scores, with individuals that are AIS A or B being more similar than those that are AIS C or D (25). Therefore, we chose to stratify randomization into the 3 arms based on AIS grade (see Table 1). We will enroll 14 participants into each arm (with equal stratification of A–B and C–D in each arm). Therefore, our target sample size to complete the entire study will be 42.

**Table 1:** Sample size and randomization cohorts.

**Arm**	**AIS A–B**	**AIS C–D**
Treatment 600 mg/day	7	7
Treatment 1,800 mg/day	7	7
Placebo	7	7

AIS, American spinal injury association impairment scale.

#### Recruitment

Because time to enrollment is short and critical, acute traumatic SCI admissions will be prescreened daily. The study physician team will be informed of all acute traumatic spinal injuries as soon as possible so they can review the medical record and discuss potential eligibility. To aid in identification and approach, the study physician team is purposefully composed of a triad specialty including neurosurgery, trauma, and physiatry. All individuals who are considered potentially eligible will be approached to discuss the study. The study was opened for enrollment on March 14, 2022. It is estimated that the final participant will be enrolled in 2026.

### Randomization and intervention

#### Allocation and blinding

Participants will be randomized to 1 of 3 groups:

- placebo,
- 600 mg/day gabapentin, or
- 1,800 mg/day gabapentin.

Group assignment will be stratified within each group based on AIS grade. A randomized stratified block design will be used with 3 blocks of size 7 within each cohort (AIS A–B and AIS C–D). A statistician will create a list under this design prior to enrollment of the first participant.

Blinded assessors will be designated for this study. The remainder of the study team will not be blinded to monitor all aspects of the trial. Blinding will be maintained by using the blinding plan described here to follow throughout the study.

- The blinded assessors will be independent from the medical team and the research staff.
- Interactions between the blinded assessors and participants will be limited.
- Blinded assessments will be performed in a private room.
- The blinded assessors will be reminded prior to each assessment of the importance of blinding and to not engaging in extra “chit chat” with participants (and family/caregivers) or other study staff.
- Participants (and family/caregivers) will be informed of the importance of blinding overall at the beginning of the study and will be reminded prior to each blinded assessment to make sure he/she does not tell the blinded assessor(s) which group they are in.
- Blinded assessors will not review any previous blinded assessments.
- Blinded assessors will review the ‘Study Blinding Checklist' immediately before each blinded exam and will complete the ‘Blinding Verification Questionnaire' immediately after each blinded exam.
- If a participant becomes unblinded to his/her treatment group, every effort will be made to keep the blinded assessors blinded.
- If a participant develops neuropathic pain during the 90-day treatment window and the clinical team decides all other neuropathic pain treatments are ineffective, impractical, or contraindicated, study drug will be stopped and standard of care gabapentinoids may be used. The participant will not be withdrawn from the study, though, unless he/she desires to be withdrawn. Study drug will no longer be given, but any remain study visits will be completed. The blinded assessors will remain blinded of all knowledge that the study drug has been stopped.

Attempts will be made to blind participants, but as described below, the placebo and gabapentin capsules will not be identical.

#### Intervention

Study medication will be started within 120 h post-injury and continued for 90 days. Both treatment groups (600 or 1,800 mg/day gabapentin) will receive 2 capsules of gabapentin by mouth 3 times per day for 90 days. Gabapentin dose will be gradually increased upon initial administration to reach the target dose and will be gradually decreased at termination (26). During acute hospitalization, gabapentin will be provided by the inpatient research pharmacy. At discharge, the study clinician will order the remaining supply from the research pharmacy and tapering instructions will be clearly provided to the participant. Participants temporarily unable to swallow pills by mouth (i.e., those required feeding tubes) will receive gabapentin dissolved in water at the same dosage.

The control group will receive 2 placebo capsules consisting of inert cellulose by mouth 3 times per day for 90 days. Placebo doses will be tapered up and down in the same manner as gabapentin. The capsules will be prepared by a compound pharmacy (Klein's Pharmacy, Cuyahoga Falls, Ohio) and will be administered by the research pharmacy. Participants unable to swallow pills by mouth will receive placebo dissolved in water.

All participants will be asked to abstain from taking additional gabapentinoids, (such as pregabalin or additional doses of gabapentin), during the 90-day treatment period. Person-specific treatment plans for neuropathic pain affecting function or quality of life will be deferred to the clinical team. No other clinical treatments will be restricted. Evidence-based alternative treatments include (but are not limited to) amitriptyline, tramadol, lamotrigine, transcutaneous electrical nerve stimulation (TENS), and oxycodone (27). If the clinical team decides all other pain treatments are ineffective, impractical, or contraindicated, the study drug will be stopped and standard of care gabapentinoids may be used.

### Assessments

After consent, randomization, and the initiation of treatment (all occurring no later than 120 h post-injury), follow-up will occur based on the schedule of visits listed in Table 2. To mimic potential timing of visits in a future efficacy trial, we included a Baseline, End of Treatment, and End of Study visit, which include the exploratory blinded efficacy and unblinded safety assessments. The timing of the Baseline visit (7 days post-injury ±2 days), which is just after start of study medication, is related to the complexity of performing outcome assessments in the immediate few days after injury. This approach has been used in several acute intervention trials in SCI (28–32). The Interim visit is included primarily to monitor pain transitions. The total length of each participant's time in the trial is 6 months.

**Table 2:** Schedule of visits.

	**Screening**	**Baseline (7 dpi+/−2d)**	**Interim (30 dpi+/−5d)**	**End of 90 day treatment(+14 d)**	**End of study (180 dpi +/−14d)**
**Consent**					
Informed consent form	X				
**Eligibility**					
Inclusion/Exclusion criteria	X				
**Exploratory blinded efficacy assessments**					
ISNCSCI		X	X	X	X
ISAFSCI		X		X	X
SCI-SET		X		X	X
SCIM III		X		X	X
ISCI QoL BDS		X		X	X
**Exploratory unblinded safety assessments**					
DN4		X	X	X	X
ISCI Pain BDS v2		X	X	X	X
Study medication log		X	X	X	
Adverse events		X	X	X	X
Concomitant medication log		X	X	X	X
Exit form					X

DPI, days post-injury; ISNCSCI, International Standards for Neurological Classification of SCI; ISAFSCI, International Standards for Autonomic Function after SCI; SCI-SET, SCI Spasticity Evaluation Tool; SCIM III, Spinal Cord Independence Measure version 3; ISCI QoL BDS, International SCI Quality of Life Basic Data Set; DN4, Douleur Neuropathique 4 Questionnaire; ISCI Pain BDS v2, International SCI Pain Basic Data Set version 2.

#### Primary feasibility assessments

The primary endpoint of this trial is feasibility. Table 3 lists the measures and quantitative benchmarks of success for the following four feasibility questions:

1. Can the target population be recruited?
2. Can the drug treatment protocol be delivered?
3. Can the assessors remain blinded?
4. Will participants complete the study?

**Table 3:** Analytical questions, measures, and benchmarks of success.

**Question**	**Measure**	**Quantitative benchmark of success**
Can the target population be recruited?	Number screened/month; number enrolled/month; reasons for not enrolling	Screen an average of 4/month; Enroll an average of 1/month
Can the drug treatment protocol be delivered?	Proportion of enrolled who receive the full drug treatment protocol (placebo arm; two gabapentin dose arms); number of dosing deviations/arm; reasons for dosing deviations	70% enrolled in placebo arm receive full dosing protocol; 70% enrolled in each gabapentin arm receive full dosing protocol
Can the assessors remain blinded?	Number of occurrences of unblinding; reasons for unblinding	5% or fewer occurrences of unblinding
Will participants complete the study?	Retention rate; reasons for dropout; proportion of planned assessments completed	70% of participants stay enrolled until the end of the study and complete 3 of the 4 assessment visits

#### Exploratory efficacy assessments

The International Standards for Neurological Classification of SCI (ISNCSCI) was selected as the most important blinded assessment of neurologic change (33) because of the emerging evidence suggesting that early gabapentin has a positive effect on motor-based neurologic recovery. An unblinded clinical assessment of neurologic level of injury will be used during screening to verify eligibility, but a blinded full ISNCSCI exam will be performed at all other visits. The ISNCSCI will provide multiple neurologic outcomes, including upper extremity motor score, lower extremity motor score, total motor score, light touch sensory score, pinprick sensory score, deep anal pressure, voluntary anal contraction, zone of partial preservation, and AIS grade data.

The International Standards for the documentation of Autonomic Function after SCI (ISAFSCI; (34, 35) was recently revised by the Autonomic Standards committee of the American Spinal Injury Association (36). As the revised ISAFSCI has not yet been validated and because emerging human data evaluating the effect of early gabapentin on autonomic function are not yet available, we chose to evaluate autonomic function in an exploratory manner. The cardiovascular, bladder, and bowel components of the revised ISAFSCI will be used. The cardiovascular component measures heart rate and blood pressure changes before and after a sit-up tilt test. Categorical scores are also assigned based on standardized definitions of autonomic responses. The bladder component measures sensation in the T11-12 and S3-5 dermatomes and motor function in the S3-5 dermatomes, which are then assigned a categorical score related to awareness of bladder fullness and ability to prevent bladder leakage. The bowel component measures the sensory and motor preservation at the S3-5 dermatomes, then assigns categorical and subjective scores related to awareness of bowel fullness and ability to prevent bowel leakage.

If early administration of gabapentin reduces maladaptive plasticity, as suggested by preclinical evidence, it may also be important to measure spasticity in a future efficacy trial (12, 13). Additionally, gabapentin has been used previously for the management of spasticity (37, 38). Spasticity is multi-faceted and can have both positive and negatives impacts on the individual. The validated Modified SCI Spasticity Evaluation Tool (SCI-SET) will be used (39). This is a self-report tool and data will be obtained through interview. The Modified SCI-SET contains 33 items that measure the positive or negative effects of spasticity on activity and participation, using a 4-level rating scale.

It will also be important in the future to link neurologic changes with functional changes. There is a known relationship between ISNCSCI motor scores and the Spinal Cord Independence Measure version 3 [SCIM III; (18, 40)]. Therefore, the SCIM III will be used as an exploratory measure of global function (41, 42). The SCIM III measures self-care, sphincter and respiration, and mobility; a score is assigned for each category of function, which is summed to generate a final score between 0 and100. SCIM III data will be obtained through interview.

Ultimately, any neurologic and functional improvement should have an impact on quality of life. For this exploratory measure, the validated International SCI Quality of Life Basic Data Set [ISCI QoL BDS; (43, 44)] was chosen. The ISCI QoL BDS is a 3-item subjective measure of an individuals' personal perspective of his/her quality of life as a whole, satisfaction with physical health, and satisfaction with psychological health. A score between 0 and 10 is assigned to each question. The ISCI QoL BDS data will be obtained through interview.

#### Unblinded safety assessments

##### Pain

As gabapentin is a first-line treatment for SCI-induced neuropathic pain and participants will be randomized to a low- or medium-dose gabapentin or placebo for the first 3 months' post-injury, it is important to track pain and its potential interference throughout the study period. The International SCI Pain Basic Data Set (ISCI Pain BDS) version 2 will be used for this as it has been well validated (45–47). Though most below-level neuropathic pains develop progressively between 1- and 12-months' post-injury, pain can develop immediately in a small subset of individuals (48). It may be necessary to exclude individuals that develop hyperacute below-level neuropathic pain from a future efficacy trial. It can be challenging to identify below-level neuropathic pain in the acute trauma setting and differentiate it from other acute pains. Therefore, in addition to the ISCI Pain BDS, we will use the Douleur Neuropathique 4 (DN4) on up to 2 below-level pain locations. The DN4 will objectively separate neuropathic pain from other pains (49, 50). The ISNCSCI data in combination with the ISCI Pain BDS can objectively determine above, at-, or below-level classification. The ISCI Pain BDS can objectively rate the pain intensity and interference. This should enable the ability to track the transition from acute to chronic pain during the 6-month study period.

##### Adverse events

It is very common for individuals that sustain a traumatic SCI to experience complications, most commonly within the first 14 days' post-injury. The most frequent types of moderate-severe complications are respiratory failure, pneumonia, pleural effusion, anemia, cardiac dysrhythmia, and severe bradycardia (51). We will track general complication adverse events (AEs) based on the following organ/systems: pulmonary, infectious, hematologic, cardiac, gastrointestinal/genitourinary, skin, and neuropsychiatric. We will also track AEs based on the known risk profile of gabapentin in SCI (dizziness, nausea, somnolence, peripheral edema, weakness/fatigue, diarrhea, constipation, blurred vision, headache, dry mouth, itching). All AEs will be evaluated for severity, expectedness, and relatedness following FDA and IRB guidelines.

A designated Medical Monitor (MM) will monitor for clinical safety. The MM will be a MetroHealth traumatologist who has extensive experience managing severely ill surgical and trauma patients in the intensive care setting and, thus, is well suited to monitor this acute intervention. AE reports will be provided to the MM on a quarterly basis. The MM will examine all AEs for reportability (unexpected, related or possibly related, AND resulting in increased risk). Non-reportable AEs will be documented internally and included in IRB continuing reviews. The study team will also have access to the MM in real time to address any urgent safety issues that may arise. A safety report will be generated annually to coincide with IRB continuing review.

Discontinuation criteria are as follows:

1. If a participant's medical condition deteriorates due to disease progression, the PI in consultation with the clinical team may withdraw the participant.
2. If a participant develops neuropathic pain during the 90-day treatment window and the clinical team decides all other neuropathic pain treatments are ineffective, impractical, or contraindicated, study drug will be stopped and standard of care gabapentinoids may be used. The participant will not be withdrawn from the study, though, unless he/she desires to be withdrawn. Study drug will no longer be given, but any remain study visits will be completed.

##### Study and concomitant medications

Important analysis criteria focus on compliance with the treatment protocol. In the inpatient setting, the study team will use the medical chart to keep a daily log of gabapentin and placebo dosing for each participant. Upon discharge, the participants, with weekly phone call assistance from the study team, will track study medications for the remainder of the study. The study team will document reasons for any deviations from the protocol-specified dosing plan. In addition, the study team will log all concomitant medications for the 6-month study period. In the inpatient setting this will be done by chart review and in the outpatient setting it will be collected during the weekly phone calls with participants. Information pertaining to the need for gabapentinoids use for pain management (if all other options fail) will be captured on the concomitant medication log and is critical for planning pain management in a future efficacy trial.

### Other considerations

As gabapentin is metabolized in the kidney, it is important to monitor renal function because some participants may have temporary renal impairment due to the initial injury that could require adjustment of gabapentin dose. All participants will undergo renal function testing as per clinical care. Testing typically occurs at the following minimum frequency while admitted to the different hospital settings: daily in the intensive care unit (ICU) acute care hospital and weekly in non-ICU acute care hospital. Testing during the inpatient rehabilitation and outpatient settings is at the discretion of the treating physician. Glomerular filtration rate (GFR) will be estimated using a dynamic formula since standard calculations are based on stable chronic disease (Physicians' Desk Reference). Study gabapentin will be renally dosed when needed (and documented). Control group medication will not be adjusted. A nephrologist will serve in an advisory role and provide input on participant safety issues.

Monitoring for other signs of poor gabapentin tolerance (such as confusion, sedation, edema, etc.) will occur at all study visits. Inpatient clinical teams will also be encouraged to report potential side effects. Gabapentin dose may be decreased by the unblinded investigators if needed.

Finally, this study using gabapentin falls under FDA Investigational New Drug (IND) exemption. Criteria for being exempt from filing an IND described under 21 CFR 312.2(b) *Exemptions* state that the clinical investigation of a drug that is lawfully marketed in the US is exempt if all the criteria in Table 4 apply.

**Table 4:** CFR 312.2(b) IND exemptions.

**Criteria**	**Rationale**
The investigation is not intended to be reported to FDA as a well-controlled study in support of a new indication for use nor intended to be used to support any other significant change in the labeling for the drug.	We are not intending to report this data to the FDA to support a new indication or a change in labeling.
If the drug that is undergoing investigation is lawfully marketed as a prescription drug product, the investigation is not intended to support a significant change in the advertising for the product.	This study would not change any advertising.
The investigation does not involve a route of administration or dosage level or use in a patient population or other factor that significantly increases the risks (or decreases the acceptability of the risks) associated with the use of the drug product.	There is a long history of using gabapentin clinically in the broad SCI population via this route of administration and dosage level as well as up to 3,600mg daily.
The investigation is conducted in compliance with the requirements for institutional review set forth in part 56 and with the requirements for informed consent set forth in part 50.	This study will be conducted under the MHS IRB oversight.
The investigation is conducted in compliance with the requirements of § 312.7. (a) Promotion of an investigational new drug. A sponsor or investigator, or any person acting on behalf of a sponsor or investigator, shall not represent in a promotional context that an investigational new drug is safe or effective for the purposes for which it is under investigation or otherwise promote the drug. This provision is not intended to restrict the full exchange of scientific information concerning the drug, including dissemination of scientific findings in scientific or lay media. (b) Commercial distribution of an investigational new drug. A sponsor or investigator shall not commercially distribute or test market an investigational new drug. (c) Prolonging an investigation. A sponsor shall not unduly prolong an investigation after finding that the results of the investigation appear to establish sufficient data to support a marketing application.	This is not a new drug, we are not promoting any claims of safety or efficacy, we are not distributing anything commercially, and the data to be generated will not support a marketing application.

### Data analysis

#### Data security

Participants will be assigned a study code upon study entry. The linkage between the study code and participant identity will be stored in a secured REDCap database. Paper consent forms will be stored in a locked cabinet in a locked room with only study staff access. The study code will be used on all case report forms. Data will be evaluated for quality assurance in real time by the Principal Investigator. After which all data will be entered into a separate secured REDCap database for storage by the study coordinator. Quality assurance procedures will be repeated for all data entered into the database.

#### Primary data analysis

Primary data analysis will be conducted based on the benchmarks listed in Table 3. If all the quantitative benchmarks of success are met, it will be considered feasible to pursue an efficacy trial without significant protocol modification to determine the efficacy of early administration of gabapentin as an intervention for neurorecovery.

#### Secondary data analysis

Additionally, we will report the population demographics, adverse events, medications, outcome measures as well as pain characteristics and transitions. To do so we will calculate descriptive statistics for each outcome (e.g., frequency counts, percentages, means, medians, standard deviations, interquartile ranges, confidence intervals). We will report overall results within each cohort (placebo, 600, 1,800 mg gabapentin) as well as differences across cohorts. We will not test for statistical significance of efficacy because that is not the intent of this study.

In addition to using the descriptive statistics, we will perform a sensitivity analysis to inform a future efficacy trial. The literature suggests using the feasibility study's upper limit of the 80% confidence interval for the standard deviation when calculating the sample size needed for the future efficacy trial (52). We will analyze the blinded ISNCSCI total motor score results with confidence intervals at 75, 80, 85, 90, and 95% to get a preliminary, cautious view of efficacy/effect (22). This thorough sensitivity analysis will inform a sample size calculation for a future trial. Calculating the eligibility rate and the retention rate from this trial will also be helpful in estimating the duration of a future efficacy trial (22).

## Discussion

Because gabapentin is already FDA approved, is available generically, and has a long history of use in SCI, it is a prime candidate for drug repurposing. The risk to benefit ratio is attractive as well. Gabapentin has been used clinically in tens of thousands if not hundreds of thousands (or more worldwide) of individuals living with SCI and the risk profile is well known and relatively well tolerated. Gabapentin has a much lower risk profile than the only other medication previously used in the acute setting to improve neurologic outcomes in SCI: high-dose methylprednisolone (53). The unknown risk relates to the fact that gabapentin is being administered slightly earlier than usual for the clinical management of neuropathic pain (starting 5 days after injury as opposed to a few weeks or months after injury). Although, gabapentin is increasingly used in the post-operative period after acute trauma for pain management.

The preclinical and clinical evidence of benefit is strong enough to warrant moving forward to the next step in translation, which is this trial. Additionally, acute interventions available for traumatic SCI are severely lacking as only early surgical decompression and targeted spinal cord perfusion pressure have demonstrated likely benefits. There is the generally accepted belief in the field that significant clinically meaningful benefit will only come from the cumulative effective of multiple interventions with each contributing a marginal neurologic benefit (54); gabapentin could be one of the multiple interventions.

### Rationale for dose

Extrapolation of the human equivalent dose (HED) from an animal dose involves allometric scaling, which takes into account interspecies differences in anatomy and physiology and scales based on body surface area and body weight (55). Table 5 shows the HED calculated for the animal studies described in the introduction. The HED ranging from 8.1 to 16.2 mg/kg had positive neurologic effects in animals; the HED of 64.8 mg/kg did not have a neurologic effect in animals. In the USA, gabapentin is commonly available in 100, 300, and 400 mg capsules. Dosages in the retrospective analyses in humans described in the introduction were not defined, but likely ranged from 300 to 3,600 mg/day. The generally effective clinical dose for neuropathic pain with minimal side effects is approximately 1,800 mg/day. Based on all this information, it was decided to explore a low (600 mg/day) and medium (1,800 mg/day) dose of gabapentin in comparison to placebo.

**Table 5:** Animal to human dose conversion.

**References**	**Species**	**Dose used and HED**	**Daily dose for 80 kg human**
Kitzman et al. (12)	Rats	50mg/kg; HED = 8.1 mg/kg	648 mg
Rabchevsky et al. (13)	Rats	50mg/kg; HED = 8.1 mg/kg	648 mg
Rabchevsky et al. (14)	Rats	50mg/kg; HED = 8.1 mg/kg	648 mg
Eldahan et al. (56)	Rats	400 mg/kg; HED = 64.8 mg/kg	5,184 mg
Sun et al. (15)	Mice	92–138 mg/kg; HED = 7.4–11.2 mg/kg	592–896 mg
Brennan et al. (4)	Mice	200 mg/kg; HED = 16.2 mg/kg	1,296 mg

### Rationale for start time

In the animal studies described above, gabapentin was started immediately after SCI or within the first day. The human literature suggests that the largest effects may be achieved when gabapentin is started within 0–5 days of injury. Therefore, the target enrollment window is as soon as possible following admission to the hospital and extending through 5 days (120 h) post-injury.

### Rationale for duration of treatment

From the animal literature, the duration of dosing ranged from 14 to 131 days post-SCI, with the average across studies being 52 days (4, 14, 15, 56). In humans, spinal shock typically ends, and early hyperreflexia emerges by 1-month post injury, with late hyperreflexia emerging between 1 and 12 months' post-injury (57). With this combined information, a 90-day treatment duration was chosen for this first prospective study.

### Input from stakeholders

Input on the topic and design of this trial was obtained from our Community Advisory Board (CAB), composed of individuals living with SCI, in an iterative manner. In the initial idea-generation phase of this trial, exceedingly helpful input was obtained from the CAB. During a preliminary meeting, they indicated their approval with use of low dose gabapentin (there was concern about side effects of high doses used in chronic pain management) and with alternative pain management options. Given a discussion about participants receiving gabapentin unnecessarily (e.g., for research), the CAB sought to ensure that people would be weaned off the study drug, which was built into the design at the end of the 90-day treatment period. To address their concern that autonomy be maintained, it was decided to include only those personally able to give informed consent during the enrollment window. The CAB did not question the inclusion of either a placebo group or dissolving the drug in water, should anyone be unable to swallow pills temporarily. Their input was incorporated into the final design of this study, which was then communicated back to the CAB and their approval was obtained.

We also sought input from MetroHealth System clinical sub-specialists that are key contributors in the clinical care for SCI and this project: Trauma, Neurosurgery, Pain Management, Nephrology, and PM&R. They all provided input to different aspects of the trial and several agreed to serve in an advisory role. Finally, we sought input from scientists external to the study team. One has published the retrospective analyses of the effect of gabapentin on neurorecovery in humans (John Kramer) and the other has published several prospective animal studies demonstrating the effect of gabapentin on the autonomic system (Alexander Rabchevsky). Both individuals agreed to serve in an advisory role throughout the trial.

### Limitations

We recognize that there are some limitations to this study protocol. One is that the placebo and gabapentin capsules are not identical. Participants could search online without the study team knowing and identify what treatment they are on based on the size, shape, color, and markings on capsules. By unblinding themselves this could create bias in the results. Also, the placebo and gabapentin capsules may taste different leading participants to identify which treatment they are on if they end up needing gabapentin for pain management after the 90-day treatment period. This could also create bias in the results.

The broad inclusion of all injury levels and severities and randomization scheme of grouping AIS A–B and AIS C–D is a limitation with regard to analyzing trends of neurologic recovery. As we are testing the feasibility of restricting/manipulating the use of gabapentin it is important that we understand the impact of that restriction for all injury levels and severities (e.g. ability to consent within the enrollment time window, potential swallowing difficulties) to be able to inform future trial enrollment criteria and randomization schema. Regarding the potential safety of gabapentin, it has been used broadly and at much higher clinical doses in all levels and severities of SCI so there is no strong rational to restrict inclusion based on injury level or severity. However, the heterogeneity of neurorecovery across injury levels and different AIS grades will have an impact on interpretability of efficacy results despite them intended to only be exploratory. A more appropriate randomization scheme for future trials may be to group AIS A by itself, AIS B–C together, and AIS D by itself.

Additionally, it is possible that even if all of the quantitative benchmarks of success are met regarding feasibility, protocol modification may be necessary to address heterogeneity of neurorecovery in a properly powered efficacy trial. We will have a strong understanding, however, of the ability to restrict the dosage of gabapentin and use a placebo for such a trial.

## Ethics and dissemination

This trial has received ethical approval from the MetroHealth System Institutional Review Board (IRB21-00609). No changes to the protocol will be implemented prior to obtaining ethical approval.

Informed consent will be obtained by the investigators and clinical research staff in consultation with the participant. As this study has an enrollment time window cut off of 120 h post-injury, the study team will identify potential participants as quickly as possible after they are admitted to the hospital following their injury. Most individuals who sustain a traumatic SCI are admitted to this site within less than 24 h of injury onset. The goal of identifying potential participants as soon as possible after admission is to allow as much time as possible for the consent process and to include family members when requested by potential participants.

Results will be disseminated through presentation at peer-reviewed academic meetings and by publication in peer-reviewed medical journals. Publications will be shared with the Model Systems Knowledge Translation Center and the National Rehabilitation Information Center. Summaries of different results will be shared with the community living with SCI as well as clinical providers as appropriate.

## Data sharing

A deidentified dataset will be shared after the end of the award. The data will be processed into a usable format and then a copy of the de-identified dataset will be transferred to the Interuniversity Consortium for Political and Social Research (ICPSR) for data sharing and long-term preservation. The data will be released to the public no later than 24 months after the award end date. The dataset will have a digital object identifier, provided by ICPSR, for future reference and citation.

## Ethics statement

The studies involving human participants were reviewed and approved by the MetroHealth System Institutional Review Board. The participants provided their written informed consent to participate in this study.

## Author contributions

KA, JW, and MK were involved in the design and conception of this trial and manuscript and critically revised the manuscript. KA, JW, and ME-A performed literature searches. JP, SD, ME-A, JF, and MS compiled the initial draft of the primary manuscript. KA and JP compiled the figures and tables. All authors gave final approval of the version to be published.

## Funding

This trial was developed under a grant from the National Institute on Disability, Independent Living, and Rehabilitation Research (NIDILRR Grant Number 90SIMS0007). NIDILRR is a Center within the Administration for Community Living (ACL), Department of Health and Human Services (HHS). The contents of this manuscript do not necessarily represent the policy of NIDILRR, ACL, HHS, and should not be assumed as endorsement by the Federal Government.

## Conflict of interest

The authors declare that the research was conducted in the absence of any commercial or financial relationships that could be construed as a potential conflict of interest.

## Publisher's note

All claims expressed in this article are solely those of the authors and do not necessarily represent those of their affiliated organizations, or those of the publisher, the editors and the reviewers. Any product that may be evaluated in this article, or claim that may be made by its manufacturer, is not guaranteed or endorsed by the publisher.
